# Microbial diversity in Antarctic Dry Valley soils across an altitudinal gradient

**DOI:** 10.3389/fmicb.2023.1203216

**Published:** 2023-07-24

**Authors:** Lefentse Mashamaite, Pedro H. Lebre, Gilda Varliero, Silindile Maphosa, Max Ortiz, Ian D. Hogg, Don A. Cowan

**Affiliations:** ^1^Department of Biochemistry, Genetics and Microbiology, Centre for Microbial Ecology and Genomics, University of Pretoria, Pretoria, South Africa; ^2^Rhizosphere Processes Group, Swiss Federal Research Institute WSL, Birmensdorf, Switzerland; ^3^Clemson University Genomics & Bioinformatics Facility, Clemson University, Clemson, SC, United States; ^4^School of Science, University of Waikato, Hamilton, New Zealand; ^5^Canadian High Arctic Research Station, Polar Knowledge Canada, Cambridge Bay, NU, Canada

**Keywords:** Antarctic microbiology, McMurdo Dry Valleys, edaphic habitats, microbial diversity, altitudinal gradients

## Abstract

**Introduction:**

The Antarctic McMurdo Dry Valleys are geologically diverse, encompassing a wide variety of soil habitats. These environments are largely dominated by microorganisms, which drive the ecosystem services of the region. While altitude is a well-established driver of eukaryotic biodiversity in these Antarctic ice-free areas (and many non-Antarctic environments), little is known of the relationship between altitude and microbial community structure and functionality in continental Antarctica.

**Methods:**

We analysed prokaryotic and lower eukaryotic diversity from soil samples across a 684 m altitudinal transect in the lower Taylor Valley, Antarctica and performed a phylogenic characterization of soil microbial communities using short-read sequencing of the 16S rRNA and ITS marker gene amplicons.

**Results and Discussion:**

Phylogenetic analysis showed clear altitudinal trends in soil microbial composition and structure. Cyanobacteria were more prevalent in higher altitude samples, while the highly stress resistant Chloroflexota and Deinococcota were more prevalent in lower altitude samples. We also detected a shift from Basidiomycota to Chytridiomycota with increasing altitude. Several genera associated with trace gas chemotrophy, including *Rubrobacter* and *Ornithinicoccus*, were widely distributed across the entire transect, suggesting that trace-gas chemotrophy may be an important trophic strategy for microbial survival in oligotrophic environments. The ratio of trace-gas chemotrophs to photoautotrophs was significantly higher in lower altitude samples. Co-occurrence network analysis of prokaryotic communities showed some significant differences in connectivity within the communities from different altitudinal zones, with cyanobacterial and trace-gas chemotrophy-associated taxa being identified as potential keystone taxa for soil communities at higher altitudes. By contrast, the prokaryotic network at low altitudes was dominated by heterotrophic keystone taxa, thus suggesting a clear trophic distinction between soil prokaryotic communities at different altitudes. Based on these results, we conclude that altitude is an important driver of microbial ecology in Antarctic ice-free soil habitats.

## 1. Introduction

The Antarctic McMurdo Dry Valleys, encompassing an area of some 4,800 km^2^ and representing approximately 95% of the ice-free non-maritime land of the continent, have been the primary target for studies of Antarctic terrestrial soil microbiology for the past half-century (Freckman and Virginia, 1998; Virginia and Wall, 1999; Babalola et al., 2009; Cary et al., 2010; Lee et al., 2012; Schwartz et al., 2014). The Dry Valleys are geologically and edaphically diverse (Bockheim, 2002; Bockheim and McLeod, 2008), and comprise a wide variety of soil habitats such as exposed mineral soils and gravels (Cowan et al., 2002, 2010), desert pavements (Delpupo et al., 2017), transiently wetted sediments (Niederberger et al., 2015), crypto-(Goordial et al., 2017; Rego et al., 2019), endo- (Walker and pace, 2007) and chasmo-lithic niches (Pointing et al., 2009) and various “plant”-associated habitats such as moss beds, cyanobacterial mats, and crustose lichens (Pannewitz et al., 2003; Green et al., 2012; Power et al., 2020).

All terrestrial Antarctic habitats are subject to a variety of ‘extreme’ abiotic factors, including extreme cold, long periods with little or no light, extreme desiccation, extreme oligotrophy and physical disturbance (Vincent, 2004; Adriaenssens et al., 2017). It is widely assumed that exposure to these conditions over very long time periods will have shaped uniquely structured and adapted soil microbial communities.

Early studies, using culture-dependent methods, identified a range of cosmopolitan genera, many from the Firmicutes and Actinobacteria phyla (Friedmann and Thistle, 1993), although subsequent culturing studies have identified numerous polar-specific species (Cowan and Tow, 2004; Aislabie et al., 2006; Bottos et al., 2014; Lambrechts et al., 2019). With the advent of molecular phylogenetic methods, it was rapidly appreciated that Dry Valley soils and other terrestrial niches harboured a wide diversity of prokaryotic phylotypes (Cary et al., 2010; Lee et al., 2012; Koo et al., 2018) many of which remain uncultured.

In the absence of higher plants, trophic structures in continental Antarctic soils are largely driven by cyanobacterial photoautotrophy (Kirby et al., 2011; Makhalanyane et al., 2015; Van Goethem and Cowan, 2019), particularly in cryptic endolithic (Rego et al., 2019; Mezzasoma et al., 2022) and hypolithic (Makhalanyane et al., 2013; De los Ríos et al., 2014; Wei et al., 2016) niches. Chlorophytes, particularly microalgae, are present, although their contribution to carbon input to soil microbial communities is unknown. The recent discovery that the microbial oxidation of atmospheric trace gases, specifically H_2_ and CO, can provide sufficient energy to support soil microbial communities (Ji et al., 2017) offers a new trophic paradigm. The capacity for this autotrophic metabolism is much more physically and phylogenetically widespread than originally thought (Ji et al., 2017), and Dry Valley soils have been shown to actively assimilate atmospheric hydrogen (Ortiz et al., 2021).

Landscape-scale phylogenetic studies have clearly shown that microbial communities in Dry Valley soils are far from homogeneous (Lee et al., 2012; Stomeo et al., 2012; Bottos et al., 2020), and that the drivers of microbial community composition are complex (Lee et al., 2012). A variety of abiotic factors, including altitude, temperature and soil nutrient status (Aislabie et al., 2006; Cowan et al., 2010; Stomeo et al., 2012; Adriaenssens et al., 2017; Bottos et al., 2020) have been implicated in microbial community assembly. While yet poorly understood, it is likely that biotic factors (particularly inter-species interactions) are also significant drivers of community structure (Hogg et al., 2006; Lee et al., 2012), and soil viruses and bacteriophages (Zablocki et al., 2014; Adriaenssens et al., 2017) may be important factors in microbial community dynamics.

Water availability in edaphic niches, a complex function of precipitation regimes, temperature and atmospheric humidity, is thought to be a key determinant of both microbial diversity and microbial functionality (Goordial et al., 2016). Altitude plays an important role in soil water availability and is implicated in microbial community composition (Lee et al., 2012; Coleine et al., 2019). Higher atmospheric relative humidities and more frequent cloud cover at high altitudes increase surface water availability, and together are probably responsible for the altitudinal distribution of crustose lichens such as *Buellia* species (Cowan et al., 2011). However, the lower mean temperatures at higher altitudes directly impact the availability of liquid water. A series of studies in the 1,677 m a.s.l. University Valley (McKay, 2009; Goordial et al., 2017) have shown that, where mean temperatures are consistently too low to melt shallow ground ice (Marinova et al., 2022), the limited availability of liquid water is a major constraint on microbial diversity (Goordial et al., 2016) and functionality (Goordial et al., 2017).

To address the effects of altitude on microbial community structure, here we assessed microbial diversity from a series of samples taken across a 684 m altitudinal gradient in the lower Taylor Valley, McMurdo Dry Valleys, Antarctica. In this study, we hypothesize that the soil microbial diversity and functional potential will significantly shift across the altitudinal transect, due to shifts in abiotic variables such as soil chemistry, temperature and water availability.

## 2. Materials and methods

### 2.1. Sample collection

**Figure 1:**
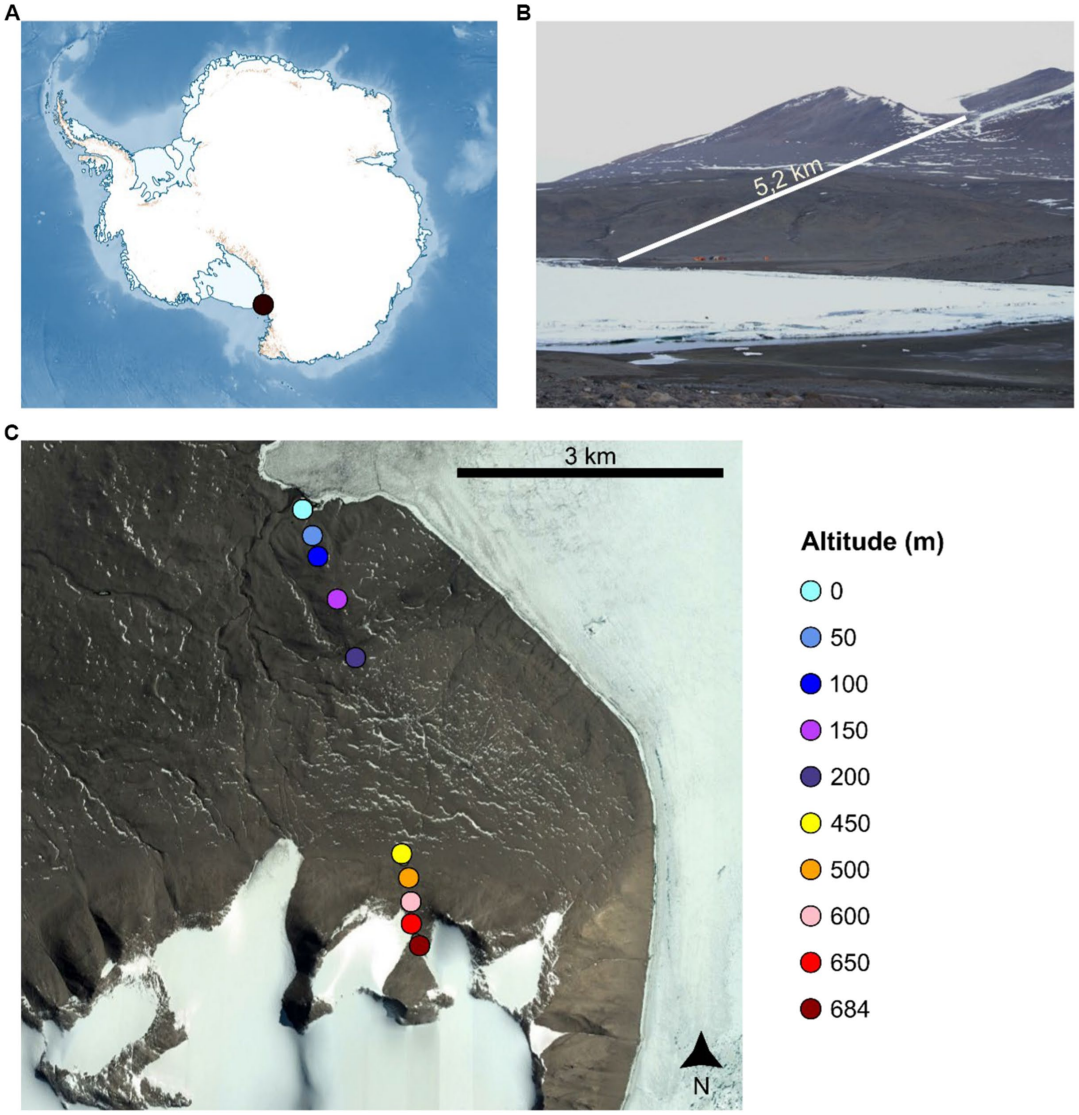
Sample location in the Antarctic continent **(A)**, and along the sampling transect in the New Harbour area (Lower Taylor Valley, McMurdo Dry Valleys, East Antarctica) **(B,C)**.

Surface (0–5 cm depth) mineral soil samples were recovered from GPS-located sites at 50 m altitudinal intervals along a *ca.* 5.2 km transect (from 0 m a.s.l. to 684 m a.s.l.) in the New Harbour area (Lower Taylor Valley, McMurdo Dry Valleys, East Antarctica) in January 2018 (Figure 1 and Supplementary Table S1). At each location, four *ca.* 200 g soil samples from sites spaced horizontally 10 m apart were recovered into sterile Whirlpaks®, where each sample was a composite of four ca. 50 g sub-samples recovered from the corners of a 1 m^2^ quadrat. All samples were retained at <0°C, transported on ice to Scott Base, Ross Island for storage at −20°C, and subsequently transported on dry ice via Christchurch, NZ to the University of Pretoria, Pretoria, South Africa where they were stored at −80°C prior to processing.

### 2.2. Soil analysis

For the soil chemistry analyses, four composite samples from each site were pooled together to generate one representative sample per site. The resulting 10 samples were analysed for geochemistry (Supplementary Table S2), including: soil pH, soil organic matter (% SOM), nitrate-nitrogen (NO_3_-N), ammonia-nitrogen (NH_4_-N) and total carbon (% C). Soil texture was also measured for % sand, % clay and % silt. Elemental analysis included K, Na, Ca, Mg, Fe, Mn, Cu, Zn, B, P and Al. All analyses were performed by NviroTek Laboratories (Hartbeespoort, South Africa).

### 2.3. DNA extraction and sequencing

DNA from the 40 samples collected from the 10 sites along the altitude gradient was extracted using DNeasy PowerSoil kits (QIAGEN, United States). DNA extraction followed the manufacturer’s instructions excepting for these modifications: 1.5 g of soil was weighed and added to the glass bead tubes for each sample; soil samples were homogenised with the PowerLyzer 24 Bench Top Bead-Based Homogenizer (QIAGEN, United States) at 4000 rpm for 1 min following the addition of solution C1. DNA quality and quantity were assessed using a NanoDrop 2000 spectrophotometer (ThermoFisher, United States) and 1% agarose gel electrophoresis. Samples were sent to Admera Biosciences (United States) for amplification and sequencing of the V3–V4 regions of the 16S rRNA gene with standard Illumina 16S primers 341F (CCTACGGGNGGCWGCAG) and 805R (GACTACHVGGGTATCTAATCC) (Herlemann et al., 2011). Similarly, for Fungi, the ITS1 and ITS4 regions were amplified using the following ITS primers; ITS1F (CTTGGTCATTTAGAGGAAGTAA) and ITS4 (TCCTCCGCTTATTGATATGC) (White et al., 1990; Gardes and Bruns, 1993). Sequencing was performed using an Illumina MiSeq instrument with 300 bp paired-end reads. Raw sequences were deposited in the European Nucleotide Archive under the accession PRJEB55870.

### 2.4. Bioinformatics and statistical analysis

Illumina adapters were removed from all 16S rRNA gene and ITS reads using trimmomatic v 0.39 (Bolger et al., 2014). Raw reads were processed in the R environment (version 4.2.2) (R Core Team, 2022) using the R library dada2 v 1.22 (Callahan et al., 2016). For the 16S rRNA gene dataset, forward and reverse reads were first quality filtered, true sequence variants were then identified in each sample, forward and reverse reads were merged into amplicon sequence variants (ASVs), and chimeric ASVs were removed. For ITS amplicons, only forward reads were used due to poor sequence quality of the reverse reads and the lack of overlap between forward and reverse reads. The ITS forward reads were treated using the same pipeline as for the 16S rRNA genes except the merging step which was not performed. Subsequently, sequences were taxonomically classified using the SILVA ribosomal RNA gene database v 138 (Quast et al., 2012) and UNITE reference database v 9.0 (Nilsson et al., 2019) for the 16S rRNA gene and ITS datasets, respectively. The number of reads in each sample is reported in Supplementary Table S3.

The ASV and taxonomy tables obtained from the dada2 pipeline were imported and analysed using phyloseq (McMurdie and Holmes, 2013). For the 16S rRNA gene dataset, only ASVs assigned to Bacteria and Archaea were retained, with the removal of ASVs assigned to mitochondria or chloroplasts. For the ITS dataset, only ASVs assigned to Fungi were retained. Only samples represented by at least 5,000 reads were retained. The dataset was then normalised using the R library SRS (Beule and Karlovsky, 2020) using the number of reads present in the smallest sample as cut-off (8,784 for 16S rRNA genes and 6,513 for the ITS dataset) (Supplementary Figure S1).

Alpha diversity was calculated using the R package vegan (Oksanen et al., 2022). Beta diversity was analysed using Bray–Curtis dissimilarity matrices obtained from the Hellinger transformed 16S rRNA gene and ITS datasets. This analysis was done by Principal Coordinates Analysis (PCoA), using ape package (Paradis and Schliep, 2019), and by performing a Permutational Multivariate Analyses of Variance (PERMANOVA) using vegan package (Oksanen et al., 2022). Plots were created using ggplot2 (Wickham et al., 2016).

Correlations between relative abundance for each phylum and altitude were calculated using the function cor.test() and then adjusting the *p* value using the False Discovery Rate (FDR) method (Benjamini and Hochberg, 1995).

The distribution of climatic and soil chemistry variables across different sites was calculated on log-standardized data using the “prcomp” function of the Vegan package, which performs a principal component analysis of the data (PCA) (Venables and Ripley, 2002). The resulting distance matrix between samples was plotted in a PCA graph, with the projected direction and magnitude of the distribution for each variable plotted in a separate loading plot. To compare samples with respect to differences in geochemical parameters, samples collected from sites between 0 and 200 m altitude are defined as “low altitude,” and those collected from sites between 450 and 684 m are defined as “high altitude” (Supplementary Table S1). The significance of the differences in geochemical variables between these two groups was calculated using PERMANOVA, with 999 permutations. Significant differences in geochemical variables between the two altitudinal groups was calculated using the Wilcoxon Rank Sum test (Wilcoxon, 1945) in the stats (version 3.6.2) package (Ripley, 2001). Significant correlations between the soil geochemical parameters and the microbial Bray-Curtis distribution across samples were estimated using the envfit() function of the Vegan package. In order for the number of samples to correspond between the microbial abundance data and soil chemistry data, ASV counts were averaged for each site. The soil chemistry variables were also tested for collinearity prior to running envfit by using the vif() function from the car (version 3.0.11) package (Fox and Weisberg, 2019), with vif values above 10 being removed.

Significant differences in relative abundances of taxa at genus-level between low and high altitude groups was inferred using ANCOMBC 1.2.2 (Lin and Peddada, 2020). ASVs were clustered into genera and absolute counts were transformed to relative abundances using the tax_glom() and transform_sample_couts() functions of the Phyloseq package, respectively. ANCOMBC ran with 1,000 max iterations, a zero_cut of 0.90, and an alpha score of 0.05. The “FDR” method was chosen for the value of p adjustments. The results were subsequently plotted as heatmaps using the pheatmap package (Kolde, 2019). As the relative abundance of different ASVs can differ by orders of magnitude, each ASV abundance was scaled individually to aid in visualising changes in ASV abundance between clusters.

Co-occurrence network analysis of prokaryotic taxa in samples belonging to the two altitudinal groups was performed using the SPIEC-EASI package (Kurtz et al., 2015), which allows for the differentiation of direct and indirect associations between taxa, and therefore minimizes the detection of spurious correlations. To further decrease network complexity and minimize spurious connections, ASVs were clustered at genus level and only taxa that were present in five or more samples within each group were considered for the analysis. The resulting networks were loaded into Gephi (v 0.92) (Bastian et al., 2009), which was used to calculate the topological features of the networks. “Hubs” were defined at the top five taxa with the highest number of connections (degrees) and highest influence on the network (betweenness centrality). To infer the importance of trace gas chemotrophs (TGCs) and phototrophs to the interactions with the soil microbial communities of the sample sites, these were manually assigned to ASVs based on their genus level taxonomy. To identify potential TGCs within our dataset, a list of genera that have been shown to have the capability to perform hydrogen oxidation was compiled from previous studies (Supplementary Table S5) (Ortiz et al., 2021; Ray et al., 2022). All ASVs belonging to the classes *Cyanobacteriia* and *Chloroflexia* were considered phototrophic. Nodes in the co-occurrence networks were coloured according to this manual annotation of the taxa regarding their trophic status. The ratio of TGCs/Phototrophs was calculated using the summed relative abundances (per sample) of taxa that were classified in the two trophic groups. The significance of the difference in trace-gas scavengers (TGC)/Phototrophs ratio between low and high altitude samples was calculated using the Wilcoxon Rank-Sum test.

## 3. Results

### 3.1. Alpha and beta diversity

**Figure 2:**
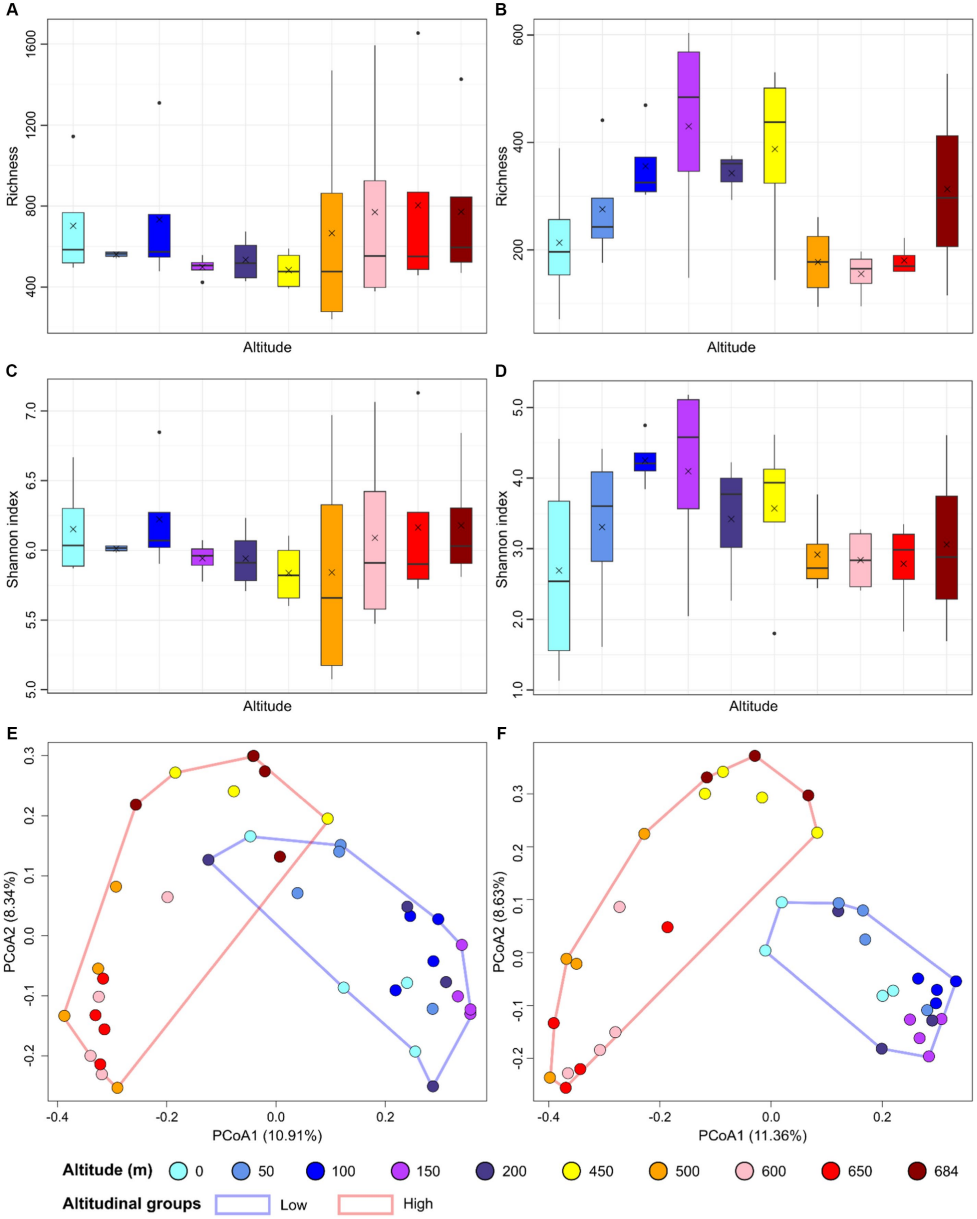
Alpha and beta diversity are represented as barplots reporting richness for bacterial **(A)** and fungal dataset **(B)**, and Shannon index for bacterial **(C)** and fungal dataset **(D)**, and principal coordinate analysis (PCoA) performed on the Hellinger-transformed ASV dataset for bacteria **(E)** and fungi **(F)**. In the boxplots, the median values are indicated with an horizontal line and the mean values with a cross for each altitude group.

Bacterial richness ranged from 240 and 1,654 in all samples (Figure 2A), whereas Shannon indices ranged from 5.1 and 7.1 (Figure 2C). Richness (*F* = 0.417, *p* = 0.916) and Shannon index (*F* = 0.343, *p* = 0.953) did not significantly differ across different altitudes for the bacterial dataset. Richness ranged from 71 to 603, and 1.1 and 5.2 for the fungal dataset (Figures 2B,D). For fungal richness, higher diversity was observed for samples at 150, 200 and 450 meters where the average richness was 430, 344, and 387, respectively. Fungal richness significantly differed for the fungal dataset (*F* = 2.373, *p* = 0.39) but not for the Shannon index (*F* = 1.013, *p* = 0.453).

The differences in community structure between samples was investigated using the Bray-Curtis distance index and Principal coordinate analysis (PCoA). Samples from the lower parts of the transect (0–200 m) formed a cluster differentiating from samples collected at higher altitudes (450–650 m) for both the prokaryotic and fungal datasets (Figures 2E,F). PERMANOVA showed an *R*^2^ of 0.1238 (*p* = 0.0009) for the bacterial dataset, and an *R*^2^ of 0.1112 (*p* = 0.0009) for the fungal dataset.

### 3.2. Microbial community composition across the altitudinal transect

**Figure 3:**
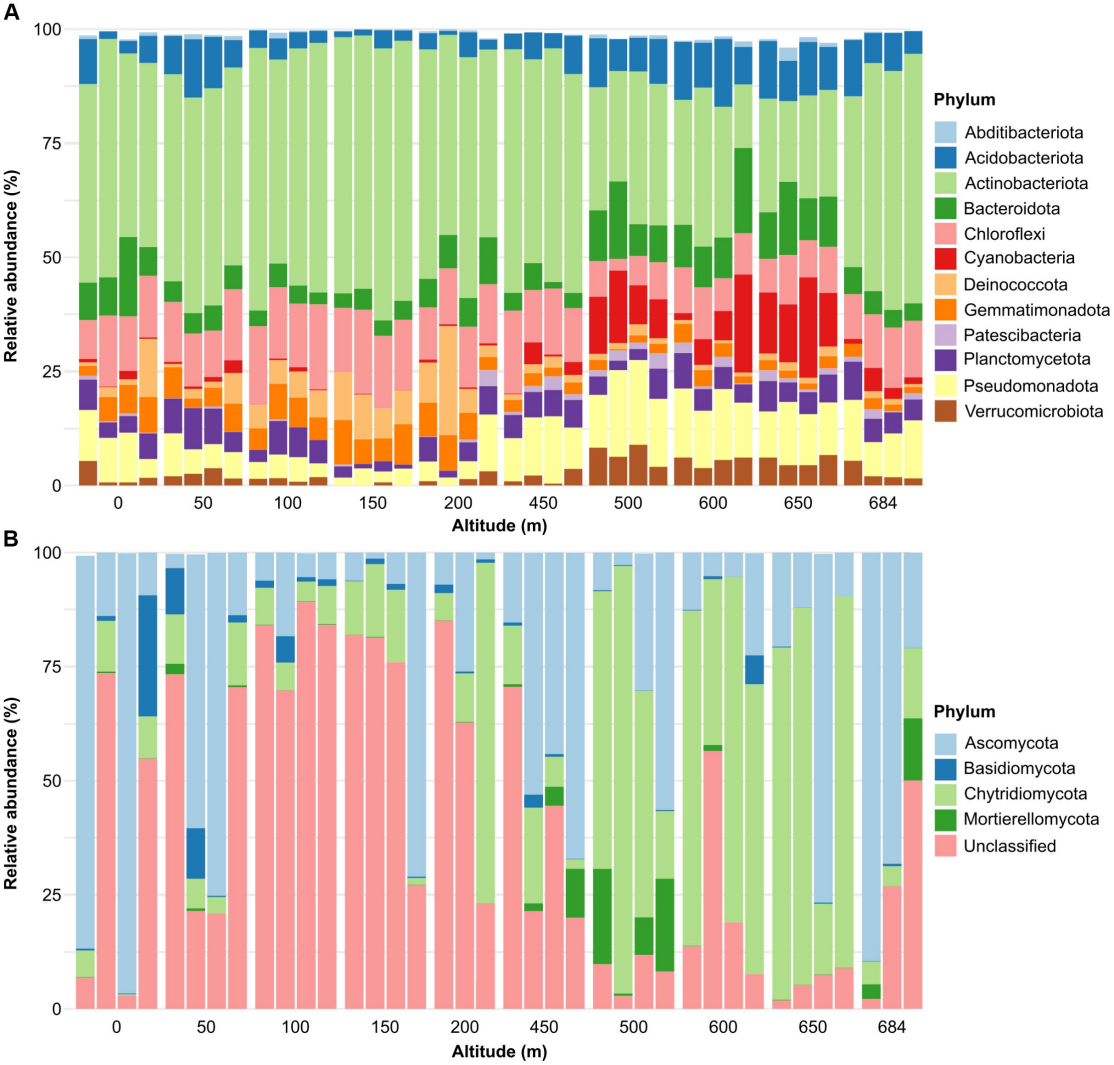
Phylum relative abundance of the bacterial **(A)** and fungal dataset **(B)**. Only dominant phyla were reported. We define dominant phyla as those present with a relative abundance higher than 1% in at least 10% of samples (i.e., four samples).

A total of 26 bacterial phyla was identified across the sample set, whereas no archaeal phyla were identified. The dominant community (i.e., phyla present with a relative abundance higher than 1% in at least 10% of samples) was composed of 12 phyla. Actinobacteria was the most dominant bacterial phylum across all the samples in the transect (13.9%–59.8%) representing 42.1% of the entire bacterial dataset, followed by Chloroflexota (12.0%), Pseudomonadota (previously Proteobacteria) (9.2%), Bacteroidota (7.2%), Acidobacteriota (6.8%), Plactomycetota (4.9%), Cyanobacteria (4.25%), Gemmatimonadota (4.1%), Deinococcota (3.8%), Verrucomicrobiota (3.0%), Patescibacteria (0.9%), and Abditibacteriota (0.5%) (Figure 3A). Five dominant phyla (Acidobacteriota, Cyanobacteria, Patescibacteria, Pseudomonadota and Verrucomicrobiota) had significant (*p* < 0.05) higher abundance in high altitude samples, whereas four dominant phyla (Actinobacteria, Chloroflexi, Deinococcota, and Gemmatimonadota) showed higher abundance in low altitude samples (Table 1). Bdellovibrionota, Elusimicrobiota, Myxococcota, Nitrospirota and SAR324 were not classified as dominant phyla but showed significant correlations between their relative abundance and altitude (Table 1). Organisms belonging to WPS-2 were present in only six samples of the dataset, with abundances ranging between 0.0% and 0.2%.

**Table 1:** Spearman’s correlations between altitude values (m) and bacterial (A) and fungal phyla (B).

Phylum	*r*	Adjusted *p*
A		
Abditibacteriota	−0.0015	0.9945
Acidobacteriota	0.4091	0.0215*
Actinobacteriota	−0.4019	0.0228*
Armatimonadota	0.3124	0.0838
Bacteroidota	0.2360	0.2026
Bdellovibrionota	0.3658	0.0421*
Chloroflexota	−0.5079	0.0032**
Crenarchaeota	0.2775	0.1319
Cyanobacteria	0.6320	0.0002**
Deinococcota	−0.4483	0.0112*
Dependentiae	0.1394	0.4591
Desulfobacterota	−0.0868	0.6685
Elusimicrobiota	0.3529	0.0492*
Firmicutes	−0.3428	0.0546
Fusobacteriota	0.1394	0.4591
Gemmatimonadota	−0.6221	0.0002**
Halobacterota	−0.1951	0.3073
Myxococcota	0.4207	0.0186*
Nitrospirota	0.6092	0.0003**
Patescibacteria	0.5681	0.0009**
Planctomycetota	0.0011	0.9945
Pseudomonadota	0.5557	0.0011**
SAR324 clade (Marine group B)	0.5098	0.0032**
Sumerlaeota	−0.1419	0.4591
Verrucomicrobiota	0.4992	0.0035**
WPS-2	−0.0146	0.9945
Unclassified	0.2527	0.1736
B		
Ascomycota	0.0751	0.9344
Basidiomycota	−0.5801	0.0013**
Blastocladiomycota	−0.1958	0.4773
Chytridiomycota	0.5141	0.0048**
Glomeromycota	−0.2807	0.2196
Monoblepharomycota	0.0301	0.9488
Mortierellomycota	0.1505	0.6120
Rozellomycota	−0.0108	0.9488
Zoopagomycota	−0.0407	0.9488
Unclassified	−0.4755	0.0085**

*p* values were adjusted using the False Discovery Rate (FDR) approach. *Adjusted *p* < 0.05. **Adjusted *p* < 0.01.

A total of nine fungal phyla were identified in the dataset. The fungal community was dominated by ASVs unclassified at the phylum-level (38.9%) (Figure 3B). The unclassified component was significantly higher in low altitude samples (*r* = −0755, *p* = 0.0085) (Table 1). The known phyla were dominated by Ascomycota, which was present in all samples across the transect (1.3%–96.4%) and represented 29.8% of the total fungal community. This was followed by Chytridiomycota which was also present in all dataset samples (0.2%–93.7%) and showed a positive significant correlation (*p* < 0.05) with altitude, being more abundant in high altitude samples (Table 1). Mortierellomycota was present with a relative abundance ranging between 0 and 20.9% of the fungal population and represented 2.4% of the total community. Basidiomycota represented 2.1% of the fungal community and showed significant (*p* < 0.05) higher abundances in low altitude samples (Table 1).

**Figure 4:**
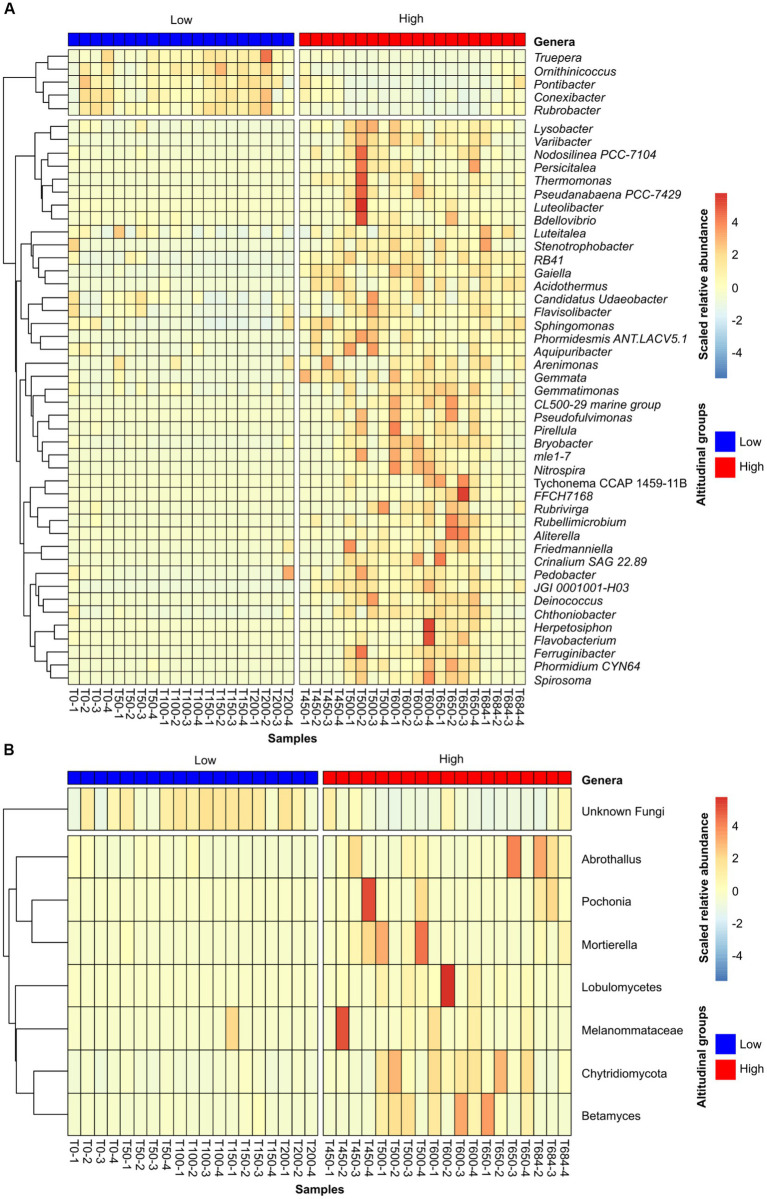
Heat maps showing the relative abundance of differentially abundant genera for the bacterial **(A)** and fungal dataset **(B)**. Samples were clustered into high/low groups according to their altitude, as established in the methods section. The heat maps only represent genera that were significantly (*p*-value < 0.05) over-represented in one of the groups, and present in at least half of the samples in the group in which they were over-represented (threshold of 10 samples for bacteria and 9 for fungi). Relative abundance values were scaled by row to emphasize the differential abundance across samples. Rows, representing taxa, were clustered according to their abundance across samples, and this clustering was visualized as dendrograms in the left-hand side of the heat maps.

The microbial composition data suggested a clear differentiation in soil microbial communities between low and high altitude samples. To further explore these differences, ANCOMBC was performed to compare the two altitudinal groups of samples in order to identify taxa at the genus level that were over-represented in either of the groups. This analysis identified a total of 126 (74 with known phylogeny to genus level) bacterial and 11 (5 known taxa) fungal taxa that were differentially represented between low and high altitudinal samples. In both cases, there was a higher number of over-represented taxa in soils from high altitude samples (Figure 4). As suggested by the correlations between phyla abundance and altitude, soils in high altitudes were enriched in photosynthetic genera, including common cyanobacterial residents in the Antarctic continent such as *Tychonema* (Salmaso et al., 2016) and *Phormidium* (Lumian et al., 2021). By comparison, low altitude soils were enriched in *Truepera*, which is a multi-stress tolerant bacterial genus (Albuquerque et al., 2005), as well as the genera *Rubrobacter* and *Ornithinicoccus*, both of which have been recently associated with the capability to scavenge trace-gases from the atmosphere in cold deserts (Ortiz et al., 2021; Ray et al., 2022) (Figure 4A). In the case of soil fungal populations, the low altitude were dominated by fungi ASVs of unknown taxonomy (Figure 4B). While not as strong, a similar, statistically significant, trend was observed for bacteria, with a higher percentage of unknown genera in soils from low altitudes (Supplementary Figure S2). Samples from high altitude sites were enriched in the lichen-associated genus *Abrothallus* (Suija, 2006), as well as saprotrophic and pathogenic fungi, including several taxa from the phylum *Chytridiomycota* (Kaczmarek and Boguś, 2021), and the nematode pathogen *Pochonia* (Ghahremani et al., 2019).

### 3.3. Soil geochemical drivers of microbial community distribution across the transect

As with the results from the microbial data, we observed a differentiation in the soil physico-chemical characteristics between the low and high altitudinal sample sites (Supplementary Figure S3): i.e., sample sites showed clear differentiation according to their geochemical composition, forming two significantly distinct clusters (*R*2 = 0.46, *p* < 0.01) for low and high altitude samples (Supplementary Figures S3A,B). The principal differences between these two groups were ammonia (NH_4_-N) and iron (Fe), which were enriched in soils at high altitude, and salts (K, Mg, Ca, Na), pH and phosphorus (P), which were higher in low altitude soils (Supplementary Figures S3A). In particular, potassium (K) and sodium (Na), which are considered indicators of proximity to coastal/marine areas, were found to be significantly enriched in soils at low altitudes (Supplementary Figures S3C,D).

**Figure 5:**
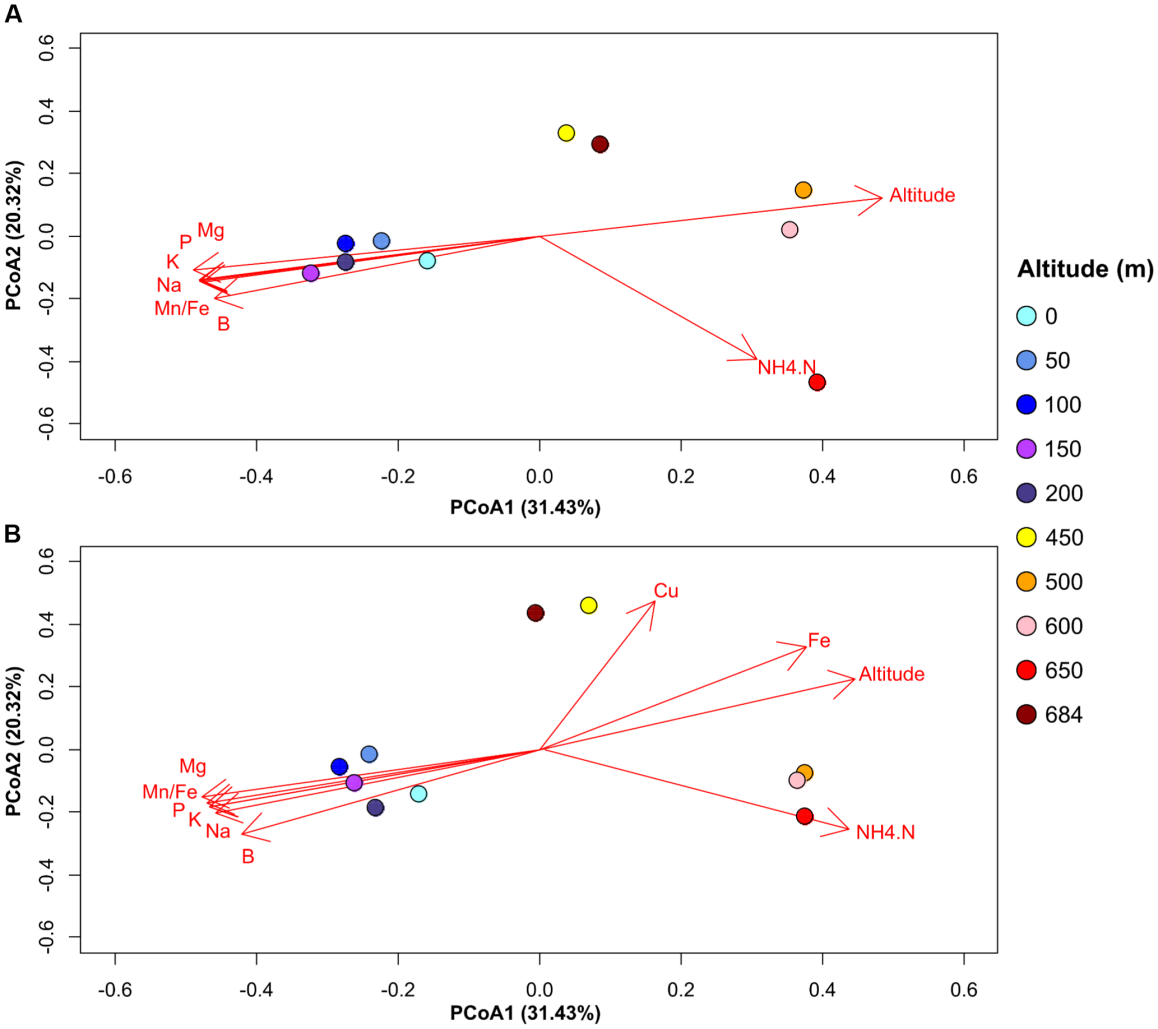
PCoA plot showing significant correlations between microbial community distribution and soil geochemical variables for bacterial **(A)** and fungal **(B)** datasets. The direction of the arrows represent the increasing trends in the values for the respective geochemical variables. Geochemical variables are represented using the following nomenclature: B—Boron (mg/kg); Cu—Copper (mg/Kg); Fe—Iron (mg/Kg); K—Potassium (mg/Kg); Mg—Magnesium (mg/Kg); Mn.Fe—ratio Manganese/Iron; Na—Sodium (mg/Kg); NH4.N—Ammonia (mg/Kg); P—Phosphorus (mg/Kg).

Taking into consideration the similarities between the microbial patterns and soil geochemical properties across the altitudinal transect, we investigated the potential soil geochemical drivers of community distribution using correlation analyses. The results show that in addition to altitude, bacterial soil community compositions across the transect were significantly correlated to NH_4_-N and phosphorus concentrations in the soil (Figure 5A). By contrast, fungal soil communities at high altitudes were positively correlated with iron and copper contents (Figure 5B). However, when using constrained analyses (db-RDA) to identify explanatory geochemical variables of soil microbial distribution, a non-significant model suggested that the geochemical data in this study lacked the statistical power to calculate reliable explanatory variables. Nonetheless, the results of the correlation analyses suggest that soil chemistry, particularly NH_4_-N and salt contents, might play a role in driving microbial community structure across the altitudinal transect.

### 3.4. Potential trophic relationships within microbial communities at different altitudes

**Figure 6:**
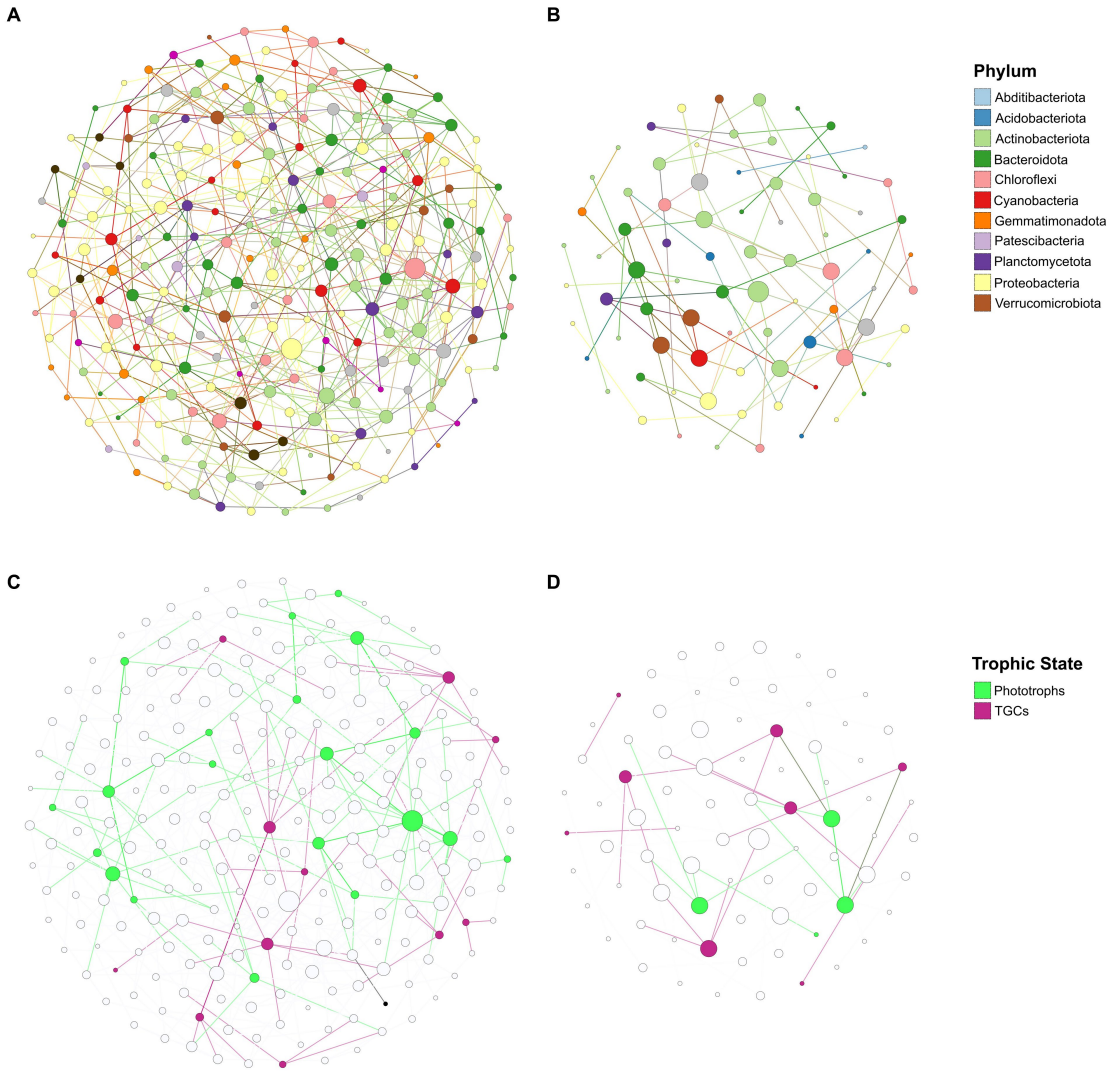
Co-occurrence networks of prokaryotic genera in high **(A)** and low **(B)** altitudinal sample groups based on their CLR-transformed abundances across samples. Node size was scaled according to degree (i.e., number of connections to other nodes) and colored according to Phylum. Edges (i.e., connections between nodes) were colored according to the source node. Nodes corresponding to phototrophs and trace-gas chemotrophs (TGC) were subsequently overlaid on the high **(C)** and low **(D)** altitudinal networks, with edges colored according to the trophic state (either TGC or phototroph) of the source node.

Co-occurrence network analysis was used to infer possible trophic relationships between taxa within soil communities in the two different elevation groups. This analysis resulted in very distinct networks across the altitudinal transect (Figure 6). Soil microbial communities from high elevation samples generated a highly connected network of 233 taxa from most of the dominant phyla in these soils, and with an average degree of 4.7 connections and a low modularity score (Modularity = 0.512) (Figure 6A). By contrast, microbial communities in low altitude samples generated a sparsely connected network with 79 taxa and an average degree of two connections, but with a higher modularity score of 0.726 (Figure 6B). We also observed large differences between the potential microbial “hubs” of the two networks, which for this study were defined as taxa with a highest number of connections (degrees) and highest influence on the network (betweenness centrality). Taking into account these two metrics, the top five hubs in the high elevation network were dominated by three Chloroflexota genera, two of which belonging to the class Chloroflexia and were therefore potentially photosynthetic (Supplementary Table S4). The top five hubs in the low altitude network consisted of mostly Actinobacteriota and Verrucomicrobiota, none of which have any predicted autotrophic capabilities based on their taxonomy (Supplementary Table S4). To further assess the possible role of trace gas chemoautotrophs and photoautotrophs in the trophic relationships of the soil communities at different altitudes, taxa in the networks were assigned as phototrophs or TGC based on a list of taxa identified in the published literature as having the genetic markers for these processes (Supplementary Table S5). Cyanobacteria and Chloroflexota phototrophs outnumbered TGCs by 20 to 11 taxa in the high elevation network, and also had an higher average degree of 5.5 (compared to 3.8 for TGCs) (Figure 6C). In the low elevation network TGC taxa outnumbered phototrophs eight to four, although phototrophs still exhibited a higher average degree (3.25 vs. 2.25) (Figure 6D). This dichotomy between phototrophs and trace gas chemotrophs across the altitudinal transect was confirmed by measuring the ratio between the relative abundances of TGCs to phototrophs in the soils (Supplementary Figure S4). TGC taxa were found to be more abundant than phototrophs across the whole dataset. However, the ratio of TGCs/Phototrophs was significantly higher in lower elevation soils (*p* < 0.05), which was in accordance with the compositional data (Figure 3), in which the percentage of Cyanobacteria was strongly correlated with elevation.

## 4. Discussion

Water, nutrient and mineral availability for microbial communities is likely to change with global warming due to shifts in climatic conditions and slow but constant melting of Antarctic ice shelves and glaciers (Gooseff et al., 2011; Rignot et al., 2013; Buelow et al., 2016). Analysing microbial community composition along altitudinal transects and coastal-inland transects, which are characterised by soil water and nutrient availability gradients (Bockheim, 2002; Fountain et al., 2010), is therefore essential to understand how microbial communities will change as a result of global warming. In our dataset, soils close to the coast showed higher concentrations of ions common in seawater (Na, Mg, and K; Supplementary Table S2 and Supplementary Figure S3), suggesting a clear input from marine sources (Millero and Huang, 2013). This trend was also observed in other coastal areas of the McMurdo Valleys (Williamson et al., 2007). Higher concentrations of P and B were also found in low altitude samples (Supplementary Table S2 and Supplementary Figure S3), which could be indicative of increased biomass closer to the coast, perhaps as biomass deposition from marine sources (Cary et al., 2010). While no moisture or temperature data was collected in this study, it is expected that water availability will increase with increasing elevation due to cloud and condensation processes (Cowan et al., 2011; Berrones Guapulema et al., 2022).

The clear differences in soil geochemistry between low and high elevation samples were also reflected in the community composition of these two groups of samples. Despite the lack of statistical robustness due to sample size (for the geochemistry analyses), our results suggest a possible correlation between the geochemistry of the transect soils and differences in community composition across the transect. For instance, the high salinity of lower altitude soils (closer to the coast) could have selected for genera such as *Rubrobacter*, *Pontibacter* and *Ornithinicoccus*, all of which are known to include extremely halotolerant species (Joshi et al., 2012; Zhang et al., 2016; Kouřilová et al., 2021). Conversely, the higher NH_4_-N concentrations (together with the likely increase in water availability) in higher altitude sites could be correlated to the enrichment of Cyanobacteria, which are known to be driven by both water and ammonia availability (Boussiba and Gibson, 1991; Herrero et al., 2001), as well as putative nitrifiers of the genus *Nitrospira* (Daims et al., 2015). While the same geochemistry variables were significantly correlated with the soil fungal distribution across the altitudinal transect, the fungal taxa that were differentially represented across the two elevation groups suggest that other factors, such as life-style and predation might also be important drivers across the altitudinal transect. In particular, the over-representation of *Chytridiomycota* taxa in the high altitude samples could be related to the over-representation of *Cyanobacteria*, as *Chytridiomycota* is the only reported fungal taxon to be able to parasitize cyanobacterial phytoplankton (Rasconi et al., 2012). Chytridiomycota have also been reported as being widely abundant in Antarctic lake systems (Ilicic et al., 2022) and are positively correlated with meltwater from glaciers (Ilicic et al., 2022). Their enrichment in the soils at high altitude, which are in close proximity to a glacial tongue (Figure 1), might therefore suggest that these soils have received a recent input from glacial meltwater. In addition, the over-representation of the lichenicolous *Ascomycota* genus *Abrothallus* in high altitude samples is consistent with the prevalence at higher altitudes of crustose lichens such as *Buellia frigida* (Sancho et al., 2019).

However, it is important to re-iterate that due to the lack of temperature and moisture data, as well as the limited number of data points, the correlations observed between soil microbial diversity and soil geochemistry as well as altitude can only be interpreted as suggestive, rather than conclusive evidence that microbial communities in this Taylor Valley transect are driven by altitude and soil chemistry. In this study, we also could not account for other processes that could play a role in the microbial diversity differences between sites: for instance the deposition of atmospheric bioaerosols (Barberán et al., 2014), legacy effects from historic ice sheet movements and water incursions (Jackson et al., 2022), as well as snow cover and snow melt cycles (Sorensen et al., 2020). Additionally, the putative role of water in the enrichment of photosynthetic taxa at higher altitudes must be treated with care, as previous water amendment studies (Van Horn et al., 2014; Buelow et al., 2016) present a very distinct shift in soil microbial diversity in response to water when compared to those shown in this study. We strongly suggest that future studies in this region of the Taylor Valley to account for soil water moisture and temperature in order to provide conclusive evidence of the role of water availability to soil microbial diversity.

The increased complexity of trophic relationships within the soil communities of high altitude soils was suggested by the co-occurrence network analysis, which predicted a more connected community in high elevation soils. The higher average degree in the high altitude network not only indicated that the members in the network were more connected than in low altitude soils, but also suggests that soil communities at high elevations are more robust; i.e. removal of one member in the community could have a smaller impact on the overall relationships between the remaining members. The higher number of connections between different phyla in the high altitude network also suggests that these communities exhibit a broader range of trophic strategies, compared to microbial communities closer to the coast. Nevertheless, putative phototrophic genera were found to be major “hubs” of the high altitude network. The “central” role of the phototrophic genera further highlights the potential importance of phototrophic microbial taxa in soil microbial communities in Antarctic soils, particularly at higher altitudes where water from cloud condensation and snowfall might reduce the xeric stress typical of Dry Valley soil habitats (Doran et al., 2002).

Trace-gas chemotrophs (TGCs) were found to be ubiquitous across the transect, which is consistent with recent studies showing their widespread distribution across many phyla and polar ecosystems (Ortiz et al., 2021; Ray et al., 2022). Despite their prevalence, these taxa were predicted to have a lower impact on the trophic relationships of the soil communities in both elevation groups compared to phototrophs, as suggested by the lower average degree of these taxa in both networks. Interestingly, TGC and phototrophic taxa were not directly connected to each other (with the exception of one connection in the low altitude network), which suggests that these two trophic strategies might not occur simultaneously. It is possible that physicochemical factors might alter the balance between co-occurring phototrophy and gas scavenging processes, but recent studies have suggested that trace-gas chemoautotrophy only becomes a dominant survival strategy in ecosystems where photosynthesis is severely constrained (Ji et al., 2017; Leung et al., 2020; Bay et al., 2021). It is important to note that due to the lack of resolution in the taxonomic assignments (taxonomy could only be assigned accurately to genus level), the trophic TGC/phototroph predictions in this study do not take into account the possibility that some phototrophs, such as specific Chloriflexota clades, might be able to perform both trophic strategies simultaneously (Islam et al., 2019), a process that has also been observed in hot desert soils after hydration (Jordaan et al., 2020). As a result, we acknowledge that our results may underestimate the relevance of trace gas chemoautotrophy to microbial communities in the altitudinal transect soils. It is also worth noting that a recent study by Dragone et al. (2022) documented an opposite trend with regards to Phototrophs/Trace-gas Chemotrophs density across different altitudes in the Shackleton Glacier region, with soils at higher altitudes exhibiting higher abundances of TGCs and lower phototrophic signals. However, these soils were characterized by lower water content and higher salt concentrations, which would correlate with the coastal sites in the present study. Thus, Dragone’s study provides further evidence that water availability might be a crucial driver of the interaction between phototrophs and TGCs in Antarctic soil communities.

In conclusion, we suggest that soil microbial communities across the elevation transect of the lower Taylor Valley are selected, through a combination of soil geochemical parameters and inputs from nearby marine and glacier sources, into distinct communities at different elevation points that exhibit distinct food-web strategies. In addition to the effects of geochemistry and elevation on soil microbial ecology, the differences in the sampling sites of this study might also reflect a long-term temporal effect in relation to inland retreat of the glacier from the coast after successive melting events (Bradley et al., 2014; Fernández-Martínez et al., 2017; Nash et al., 2018), particularly as the last major glacial retreat in the McMurdo Dry Valleys would have followed the most recent glacial maximum (*ca.* 20 ka ago: Peltier and Fairbanks, 2006). As indicated above, future studies should involve a more comprehensive sampling strategy and expand the methodology to more comprehensively measure the possible factors (including water moisture and temperature) that might drive soil microbial diversity and functional potential in Antarctic Dry Valley soils.

## Data availability statement

The datasets presented in this study can be found in online repositories. The names of the repository/repositories and accession number(s) can be found at: https://www.ebi.ac.uk/ena, PRJEB55870.

## Author contributions

DC conceptualized the study, collected the samples, supervised the analysis of the results, and contributed to writing and editing of the manuscript. IDH helped with the study conceptualization and sample logistics, as well as contributed to the final editing of the manuscript. LM was responsible for the sample and data processing, and conducted the bulk of the analyses. MO supervised the sample and data processing, and contributed to the editing of the final manuscript. PHL, GV, and SM were involved in the second round of data curation and analyses, and contributed equally to the writing of the manuscript. All authors contributed to the article and approved the submitted version.

## Conflict of interest

The authors declare that the research was conducted in the absence of any commercial or financial relationships that could be construed as a potential conflict of interest.

The reviewer BA declared a past co-authorship with the author IDH to the handling editor at the time of review.

## Publisher’s note

All claims expressed in this article are solely those of the authors and do not necessarily represent those of their affiliated organizations, or those of the publisher, the editors and the reviewers. Any product that may be evaluated in this article, or claim that may be made by its manufacturer, is not guaranteed or endorsed by the publisher.
